# The process $gg\rightarrow h^0 Z^{*}$ in the inverted hierarchy scenario of the 2HDM type-I at the LHC

**DOI:** 10.1140/epjc/s10052-024-13548-1

**Published:** 2024-11-18

**Authors:** A. G. Akeroyd, S. Alanazi, S. Moretti

**Affiliations:** 1 School of Physics and Astronomy, University of Southampton, Highfield, Southampton, SO17 1BJ UK; 2 Physics Department, Imam Mohammad Ibn Saud Islamic University (IMISU), P.O. Box 90950, 11623 Riyadh, Saudi Arabia; 3 Department of Physics and Astronomy, Uppsala University, Box 516, 751 20 Uppsala, Sweden

## Abstract

While searching at the Large Hadron Collider (LHC) for the production and decay of the CP-odd scalar ($A^0$) in the 2-Higgs-Doublet Model (2HDM) with Natural Flavour Conservation (NFC) via the channels $gg\rightarrow A^0$ (through one-loop triangle diagrams) and $A^0\rightarrow h^0 Z^*$ (with $m_{h^0} =125$ GeV or $m_{h^0} < 125$ GeV, with *Z* off-shell), respectively, a factorisation of the two processes is normally performed, with the $A^0$ state being on-shell. While this approach is gauge-invariant, it is not capturing the presence of either of the following two channels: $gg\rightarrow Z^*\rightarrow h^0Z^*$ (through one-loop triangle diagrams) or $gg\rightarrow h^0Z^*$ (through one-loop box diagrams). As the resolution of the $A^0$ mass cannot be infinitely precise, we affirm that all such contributions should be computed simultaneously, whichever the $h^0$($Z^{*}$) decay(splitting) products, thereby including all possible interferences amongst themselves. The cross section of the ensuing complete process is significantly different from that obtained in the factorisation case, being of the order up to ten percent in either direction at the integrated level and larger (including changes in the shape of kinematical observables) at the differential level. We thus suggest that the complete calculation ought to be performed while searching for $A^0$ in this channel. We illustrate this need for the case of a 2HDM of Type-I in the inverted hierarchy scenario with $m_{h^0}<125$ GeV.

## Introduction

While the discovery in 2012 of a neutral spin-0 object, consistent with the Higgs boson predicted within the Standard Model (SM), by the ATLAS and CMS collaborations of the LHC [1, 2] has signified the consolidation of this theoretical framework, despite the many measurements of its properties (mass, width, spin, charge, CP quantum numbers, etc.), it has also done very little to clarify what Beyond the SM (BSM) physics may exist in Nature. In fact, we know that some form of BSM physics must exist to remedy both theoretical (e.g., the hierarchy problem) and experimental (e.g., neutrino masses, dark matter, baryonic asymmetry of the universe) flaws of the SM.

Indeed, whether or not the observed new boson (with a measured mass of about 125 GeV) is the (lone) Higgs state of the SM or else the first manifestation of an enlarged Higgs sector is still an issue that needs to be clarified. In particular, it is possible that such a particle belongs to a 2HDM [3–6], in which the scalar potential contains two $SU(2)_L\otimes U(1)_Y$ isospin doublets instead of just one like in the SM. Herein, there exists a so-called “alignment limit”, wherein one of the CP-even scalars (of the two predicted) has properties that exactly match those of the Higgs boson of the SM. This condition is naturally obtained if all other (neutral) Higgs states have masses that are much larger than 125 GeV (the so-called “alignment with decoupling” limit). Such an alignment condition, though, can also be realised if all such (neutral) Higgs states have masses of the order of the Electro-Weak (EW) scale (the so-called “alignment without decoupling” limit). The latter will be the focus of this work.

In a 2HDM there are two CP-even (scalars) states, $h^0$ and $H^0$ (with $m_{h^0} < m_{H^0}$), a charge conjugate pair of charged states, $H^+$ and $H^-$, and a neutral CP-odd (pseudoscalar) state, $A^0$. The discovered 125 GeV boson has been shown to be neutral and CP-even. Consequently, in the context of the 2HDM, it could be interpreted as being either $h^0$ (a Higgs mass configuration called “normal hierarchy” (NH)) or $H^0$ (a Higgs mass configuration called “inverted hierarchy” (IH)). This work will consider the latter scenario.

The CP-odd $A^0$ does not have tree-level couplings to the gauge bosons of the weak interactions $(W^\pm , Z$) and is thus inconsistent with the measured properties of the discovered Higgs state. However, it could be the mediator of a signal which may reveal the IH scenario, as it can interact with both the $h^0$ and $H^0$ states, via the production and decay processes $gg\rightarrow A^0\rightarrow h^0Z^*$ and $gg\rightarrow A^0\rightarrow H^0Z^*$, respectively, where the neutral massive EW gauge boson can be considerably off-shell. In the IH scenario, the cross section for the first process is naturally unsuppressed as it is proportional to $|\cos (\beta -\alpha )|^2$, where $\alpha$ is the mixing angle in the CP-even neutral Higgs sector while $\beta$ is the arc tangent of the ratio of the Vacuum Expectation Values (VEVs) of the two Higgs doublets, which is the same coupling strength entering the $H^0VV$ (where $V=W^\pm , Z$) vertex, which has been measured to be close to 1. The second process is naturally small, as it is proportional to $|\sin (\beta -\alpha )|^2$. Therefore, it is entirely possible that $gg\rightarrow A^0\rightarrow h^0Z^*$ is discovered before $gg\rightarrow A^0\rightarrow H^0Z^*$, leading to the simultaneous discovery of two new Higgs states ($A^0$ and $h^0$).

We shall therefore focus on the prospects of discovering an $A^0$ from the 2HDM at the LHC via its production and decay process $gg\rightarrow A^0\rightarrow h^0Z^{*}$ in the context of IH but, unlike most literature, we will refrain from computing such a process on its own with the $A^0$ state treated in Narrow Width Approximation (NWA). Instead, we compute the full $gg\rightarrow h^0Z^*$ process, wherein, alongside the above channel (but modelled using a finite value of $\Gamma _{A^0}$), we also consider $gg\rightarrow Z^*\rightarrow h^0Z^{*}$ and all other diagrams entering (gauge-invariantly) the process $gg\rightarrow h^0Z^{*}$, including all interferences. We shall see that such a treatment will lead to a non-negligible modification of the sensitivity obtained using the $A^0$ diagram (only) in NWA. Thus, our present work expands and surpasses what we did in Ref. [7], where we studied the factorised process in NWA. Also, our results are corroborated by what some of us noted in Ref. [8], where effects of the additional topologies, with respect to that of the factorised process, were found to be sizeable, although that paper was concerned with a NH scenario in a so-called 2HDM Type-II, whereas we will be studying here a 2HDM Type-I in IH. (See later on for a definition of (Yukawa) ‘Types’ in the context of the 2HDM.) The interest in this particular Type of 2HDM and in such a mass spectrum comes from the fact that, herein, the decay $A^0 \rightarrow h^0 Z^*$ can have a large Branching Ratio (BR), unlike all other Yukawa configurations [7], including the popular Type-II case [8] (which can be of supersymmetric origin).

This work is organised as follows. In Sect. 2 we introduce the 2HDM that we will be using. In Sect. 3 we discuss its available parameter space in the light of current theoretical and experimental constraints. Then, in Sect. 4, we present our results. Finally, our conclusions are given in Sect. 5.

## The 2HDM

The Higgs mechanism can also be implemented using 2 complex scalar doublets for which there are now two VEVs ($v_1$ and $v_2$) and such a scenario is called the 2HDM [3–6]. Herein, after EW Symmetry Breaking (EWSB), there are 5 physical Higgs bosons remaining, as mentioned in the Introduction. In the context of the 2HDM, the 125 GeV boson discovered at the LHC is interpreted as being either $h^0$ (NH) or $H^0$ (IH), with couplings very close to those of the SM Higgs boson. The second configuration leads to interesting new phenomenology, as there would be at least one Higgs state lighter than 125 GeV. However, enlarging the scalar sector of the SM can conflict with experimental data. Firstly, a strong suppression from Flavour Changing Neutral Currents (FCNCs) data implies stringent constraints on the Yukawa structure of the 2HDM. In fact, the Yukawa couplings in the 2HDM are not flavour diagonal, in turn leading to potentially large Higgs mediated FCNCs, which must then be suppressed. A particularly elegant suppression mechanism of FCNCs in the 2HDM is invoking NFC, by exploiting the Paschos–Glashow–Weinberg theorem [9, 10], requiring that the Lagrangian respects certain discrete ($Z_2$) symmetries. Such symmetries enforce that a given flavour of charged fermion receives its mass from just one VEV, in turn leading to the elimination of FCNC processes at the tree level. In reality, such a $Z_2$ symmetry can be softly broken while still complying with the aforementioned data: this allows for more freedom in achieving EWSB.

The most general scalar potential of a 2HDM that is invariant under the $SU(2)_L\otimes U(1)_Y$ local gauge symmetry and which breaks (via the $m^2_{12}$ term) the $Z_2$ symmetry only softly is written as [4, 5]:

1 $$\begin{aligned} V(\Phi _{1}\Phi _{2})= &   m_{11}^{2}\Phi _{1}^{\dagger }\Phi _{1}+m_{22}^{2}\Phi _{2}^{\dagger }\Phi _{2}\nonumber \\  &   - m_{12}^{2}(\Phi _{1}^{\dagger }\Phi _{2}+\Phi _{2}^{\dagger }\Phi _{1})+ \frac{ \lambda _{1}}{2}(\Phi _{1}^{\dagger }\Phi _{1})^{2}\nonumber \\  &   + \frac{ \lambda _{2}}{2}(\Phi _{2}^{\dagger }\Phi _{2})^{2}+ \lambda _{3}\Phi _{1}^{\dagger }\Phi _{1}\Phi _{2}^{\dagger }\Phi _{2}\nonumber \\  &   + \lambda _{4}\Phi _{1}^{\dagger }\Phi _{2}\Phi _{2}^{\dagger }\Phi _{1}+\frac{ \lambda _{5}}{2}[(\Phi _{1}^{\dagger }\Phi _{2})^{2}+(\Phi _{2}^{\dagger }\Phi _{1})^{2}], \nonumber \\ \end{aligned}$$

with $$\Phi _{i}=\left( {\begin{array}{c}\Phi _{i}^{\dotplus }\\ \frac{(\upsilon _{i}+\rho _{i}+i\eta _{i})}{\sqrt{2}}\end{array}}\right) \,\textrm{and} \; i=1,2$$.

In general, some of the parameters in such a potential can be complex and thus they can generate CP violating effects, both spontaneously (via complex VEVs) or explicitly (through the coefficients of the operators in Eq. (1)). Here, we consider a simplified scenario by taking all such parameters to be real, as is often done in phenomenological studies of the 2HDM. The scalar potential then has 8 real independent parameters: $m^2_{11}$, $m^2_{22}$, $m^2_{12}$, $\lambda _{1}$, $\lambda _{2}$, $\lambda _{3}$, $\lambda _{4}$ and $\lambda _{5}$. These parameters determine the masses of the Higgs bosons and their couplings to fermions, gauge bosons and amongst themselves. However, it is convenient to work with different independent parameters that are more directly related to physical observables. A common choice is: $m_{h^0}$, $m_{H^0}$, $m_{H^\pm }$, $m_{A^0}$, $\upsilon _1$, $\upsilon _2$, $m^2_{12}$ and $\sin (\beta -\alpha )$. The first four parameters are the masses of the physical Higgs bosons. The VEVs $\upsilon _1$ and $\upsilon _2$ are the values of the neutral CP-even fields in $\Phi _1$ and $\Phi _2$, respectively, at the minimum of the scalar potential:

2 $$\begin{aligned} \left<\Phi _{1} \right> =\frac{1}{\sqrt{2}}\left( {\begin{array}{c}0\\ \upsilon _{1}\end{array}}\right) \,,\, \, \, \, \left<\Phi _{2} \right> =\frac{1}{\sqrt{2}}\left( {\begin{array}{c}0\\ \upsilon _{2}\end{array}}\right) . \end{aligned}$$

The parameter $\beta$ is defined via $\tan \beta =\upsilon _2/\upsilon _1$ while the angle $\alpha$ determines the composition of the CP-even mass eigenstates $h^0$ and $H^0$ in terms of the original neutral CP-even fields that are present in the isospin doublets $\Phi _1$ and $\Phi _2$. Of these 8 parameters in the scalar potential, 2 have now been measured. Firstly, after EWSB in the 2HDM, the mass of the $W^\pm$ boson is given by $m_W=gv/2$, with $\upsilon =\sqrt{\upsilon ^{2}_{1}+\upsilon ^{2}_{2}}\simeq 246$ GeV, hence, only one of $\upsilon _1$ and $\upsilon _2$ is independent ($\tan \beta =\upsilon _2/\upsilon _1$ is therefore taken as an independent parameter). Secondly, in the 2HDM, the discovered 125 GeV boson is taken to be $h^0$ or $H^0$ and thus either $m_{h^0}=125$ GeV (NH) or $m_{H^0}=125$ GeV (IH) is fixed. The remaining 6 independent parameters in the 2HDM scalar potential are therefore: $m_{H^\pm }$, $m_{A^0}$, $m^2_{12}$, $\tan \beta$, $\sin (\beta -\alpha )$ and one of $[m_{h^0}, m_{H^0}]$. In the NH scenario $m_{H^0}>125$ GeV and in the IH scenario $m_{h^0}<125$ GeV. As intimated, in this work we shall be focussing on the IH scenario and study the phenomenology of the $A^0$ and $h^0$ states in relation to each other.

As mentioned above, the masses of the pseudoscalar $A^0$ and charged $H^{\pm }$ are independent inputs parameters. In terms of the original parameters in the scalar potential, these masses are given by:

3 $$\begin{aligned} m_{A^0}^{2}&= \left[ \frac{ m_{12}^{2}}{\upsilon _{1}\upsilon _{2}} -2\lambda _{5}\right] (\upsilon _{1}^{2}+\upsilon _{2}^{2}),\nonumber \\ m_{H^{\pm }}^{2}&= \left[ \frac{ m_{12}^{2}}{\upsilon _{1}\upsilon _{2}} -\lambda _{4} -\lambda _{5}\right] (\upsilon _{1}^{2}+\upsilon _{2}^{2}) = \left[ m_{A}^{2} +\upsilon (\lambda _{5}-\lambda _{4})\right] .\nonumber \\ \end{aligned}$$

From these equations, it can be seen that the mass difference $$m_{A^0}-m_{H^\pm }$$ depends on $$\lambda _5-\lambda _4$$. In our numerical analysis, we shall be taking $$m_{A^0}=m_{H^\pm }$$ in order to satisfy easily the constraints from EW Precision Observables (EWPOs), including the so-called ‘oblique parameters’, which corresponds to $$\lambda _5=\lambda _4$$, and scan these two (equal) masses between 140 and 170 GeV. For the masses of the CP-even scalars, as mentioned earlier, we take $$m_{H^0}=125$$ GeV and $$ m_{h^0} < 125$$ GeV (IH scenario).

There are four distinct types of 2HDM with NFC which differ in how the two doublets are coupled to the charged fermions. These choices are referred to as follows: Type-I, Type-II, Lepton Specific and Flipped [11]. The Lagrangian terms in the 2HDM that describe the Yukawa interactions of $A^0$ with the fermions can be written as [5]:

4 $$\begin{aligned} {{\mathcal {L}}}^{\textrm{Yukawa}}_{A^0} =\frac{i}{v}\left( y^d_{A^0} m_d A^0 {\overline{d}} \gamma _5 d + y^u_{A^0} m_u A^0 {\overline{u}} \gamma _5 u+ y^l_{A^0} m_l A^0 \overline{l} \gamma _5 l \right) . \nonumber \\ \end{aligned}$$

In Eq. (4), *d*(*u*)[*l*] refers to the down(up)-type quarks[leptons], i.e., there are three terms of the form $y^d_{A^0} m_d {\overline{d}} \gamma _5 d$. In Table 1, the couplings $y^d_{A^0}$, $y^u_{A^0}$ and $y^l_{A^0}$ of the $A^0$ state to the charged fermions in each of the four Types are displayed.

**Table 1:** The couplings $y^d_{A^0}$, $y^u_{A^0}$, and $y^l_{A^0}$ in the Yukawa interactions of $A^0$ in the four versions of the 2HDM with NFC

	$y^d_{A^0}$	$y^u_{A^0}$	$y^l_{A^0}$
Type-I	$-\cot \beta$	$\cot \beta$	$-\cot \beta$
Type-II	$\tan \beta$	$\cot \beta$	$\tan \beta$
Lepton Specific	$-\cot \beta$	$\cot \beta$	$\tan \beta$
Flipped	$\tan \beta$	$\cot \beta$	$-\cot \beta$

## Parameter space

The viable parameter space in a 2HDM must respect all theoretical and experimental constraints, which are listed below.

1. Theoretical constraints
  (i) Vacuum stability
    The values of $\lambda _i$ are constrained by the requirement that the scalar potential: a) breaks the EW symmetry $SU(2)_L\otimes U(1)_Y$ to $U(1)_Q$, b) the scalar potential is bounded from below and c) the scalar potential stays positive for arbitrarily large values of the scalar fields. The constraints are: $\lambda _{1}> 0$, $\lambda _{2}> 0$, $\lambda _{3}+\lambda _{4}-\left| \lambda _{5} \right| + \sqrt{\lambda _{1} \lambda _{2}} \ge 0, \;\;\lambda _{3}+ \sqrt{\lambda _{1}\lambda _{2}}\ge 0$. From these conditions it can be seen that $\lambda _1$ and $\lambda _2$ are positive definite while $\lambda _3, \lambda _4$ and $\lambda _5$ can have either sign.
  (ii) Perturbativity
    For calculational purposes it is required that the quartic couplings $\lambda _{i}$ do not take numerical values for which the perturbative expansion ceases to converge. The couplings $\lambda _{i}$ remain perturbative up to the unification scale if they satisfy the condition $\left| \lambda _{i}\right| \le 8\pi$.
  (iii) Unitarity
    The $2\rightarrow 2$ scattering processes ($s_1s_2\rightarrow s_3s_4$) involving only (pseudo)scalars $s_i$ (including Goldstone bosons in the generic $R_\xi$ gauge) are mediated by scalar quartic couplings, which depend on the parameters of the scalar potential. Tree-level unitarity constraints require that the eigenvalues of the scattering matrix of the amplitudes for $s_1s_2\rightarrow s_3s_4$ be less than the unitarity limit of $8\pi$, which leads to further constraints on $\lambda _i$.
2. Experimental constraints
  (i) Direct searches for Higgs bosons
    The observation of the 125 GeV boson at the LHC and the non-observation of additional Higgs bosons at LEP, Tevatron and LHC rule out regions of the parameter space of a 2HDM. In our numerical results, these constraints are respected by using the publicly available codes HiggsBounds [12] (which implements searches for additional Higgs bosons) and HiggsSignals [13] (which implements the measurements of the 125 GeV boson). Any point in the 2HDM parameter space that violates experimental limits/measurements concerning Higgs bosons is rejected.^1^
  (ii) Oblique parameters
    The Higgs bosons in a 2HDM give contributions to the self-energies of the $W^\pm$ and *Z* bosons. The oblique parameters *S*, *T* and *U* [15], part of the aforementioned EWPOs, describe the deviation from the SM prediction of $S=T=U=0$. The current best-fit values (not including the recent CDF measurement of $m_W$ [16]) are [17]:
    5 $$\begin{aligned}  &   S=-0.01\pm 0.10,\;\; T=0.03\pm 0.12,\nonumber \\  &   U=0.02\pm 0.11 . \end{aligned}$$
    If $U=0$ is taken (which is approximately true in any 2HDM Type), then the experimentally allowed ranges for *S* and *T* are narrowed to [17]:
    6 $$\begin{aligned} S=0.00\pm 0.07,\;\; T=0.05\pm 0.06. \end{aligned}$$
    In our numerical results, the theoretical constraints in 1(i)–(iii) and the experimental constraints 2(ii) (using the ranges for *S* and *T* in Eq. (6)) are respected by using 2HDMC [18]. If the recent measurement of $m_W$ by the CDF collaboration [16] is included in the world average for $m_W$, then the central values of the *S* and *T* parameters in Eq. (6) change significantly and can be accommodated in a 2HDM by having sizeable mass splittings among the Higgs bosons. Recent such studies have been carried out in [19, 20] in both NH and IH.
  (iii) Flavour constraints
    The parameter space of a 2HDM is also constrained by flavour observables, especially the decays of *b*-quarks (inside *B*-mesons). The main origin of such constraints is the fact that the charged Higgs boson $H^\pm$ contributes to processes that are mediated by a $W^\pm$, leading to constraints on the parameters $m_{H^\pm }$ and $\tan \beta$. The flavour observable that is most constraining is the rare decay $b\rightarrow s\gamma$, although $H^\pm$ contributes to numerous other processes (e.g., $B{\overline{B}}$ mixing). There have been many studies of flavour constraints on the parameter space of the 2HDM, see, e.g., [21–23]. In our numerical analysis, we respect such flavour constraints by using the publicly available code SuperIso-v1.4 [24] and incorporating the latest values for the following three experimental measurements on BRs: BR$(B_{d}\rightarrow \mu ^{+}\mu ^{-})=3.9\times 10^{-4}$ [25], BR$(B_{s}\rightarrow \mu ^{+}\mu ^{-})= 3\times 10^{-9}$ [26] and BR$(B\rightarrow X_{s} \gamma )= 3.3\times 10^{-3}$ [27]. In the 2HDM Type-I, in which the couplings of $H^\pm$ to fermions are proportional to $\cot \beta$, the constraint on $m_{H^\pm }$ is weaker with increasing $\tan \beta$. The lowest value of $\tan \beta$ we consider is $\tan \beta =2.5$, for which $m_{H^\pm }=140$ GeV is allowed (as can be seen in [21]).

**Fig. 1:**
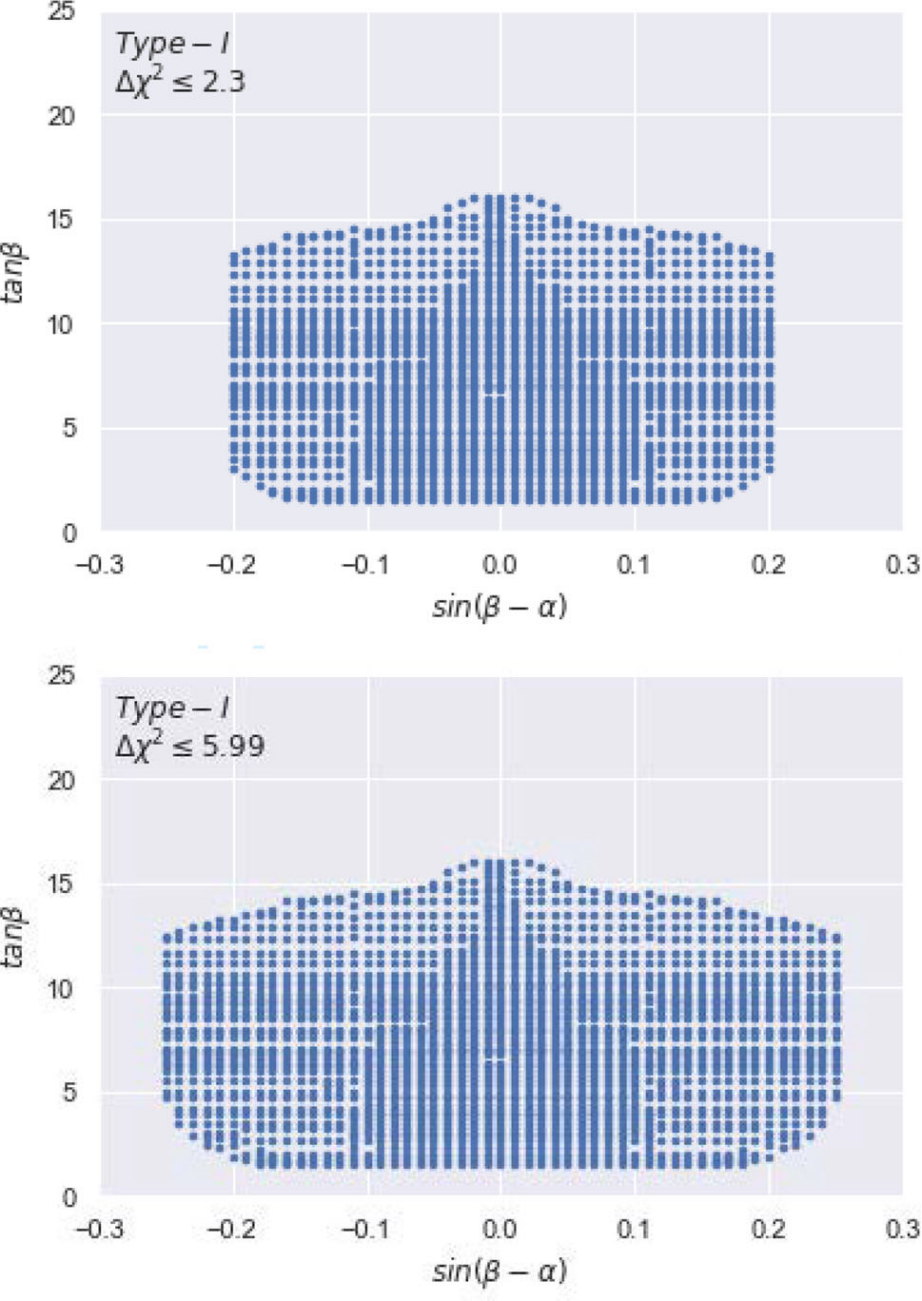
The scatter plots of the parameter space points surviving all theoretical constraints as well as all experimental ones, the latter within $1\sigma$ (top) and $2\sigma$ (bottom), mapped onto the ($\sin (\beta -\alpha ),\tan \beta$) plane for the 2HDM Type-I in IH configuration

We end this section by presenting Fig. 1, describing the parameter space of the 2HDM Type-I in IH configuration after all aforementioned constraints have been applied, as an update to Fig. 1 of Ref. [28].

## Results

**Fig. 2:**
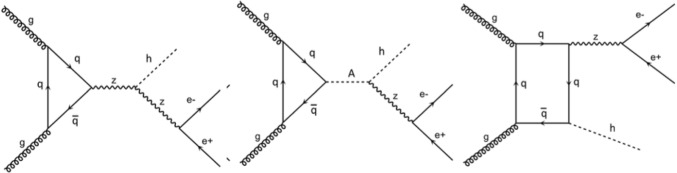
Representative Feynman diagrams for $g g\rightarrow h^{0} Z^*\rightarrow h^{0}e^{+}e^{-}$. (Note that the $b{\bar{b}}$ induced diagrams are negligible in the 2HDM Type-I in IH, so we ignore them throughout.) The summation is intended on all quark flavours, so that, in our BSM scenario, the first topology is dominated by *d*, *u*, *s*, *c* and *b* loops while the last two topologies are dominated by *t* loops

Based on the complete gauge-invariant set of diagrams to which those in Fig. 2 belong (hereafter, when calculating $Z^*\rightarrow l^+l^-$, we will be summing over $l=e,\mu$), we have computed both the integrated and differential cross section for the process $g g \rightarrow h^{0} Z^*\rightarrow h^{0}l^{+}l^{-}$ at Leading Order (LO) with MadGraph5aMC@NLO [29], with default Parton Distribution Functions (PDFs) and corresponding factorisation/renormalisation scale.^2^ Then we have used MadAnalysis [37] to analyse Monte Carlo (MC) partonic events, specifically, in looking at the invariant mass of the system $h^{0}l^{+}l^{-}$ for a small sample of pseudo-randomly generated parameter space points, over the interval 140 GeV $<m_{A^{0}}<$ 170 GeV, with the mass of the charged Higgs boson set equal to the mass of the CP-odd Higgs boson (i.e., $m_{H^\pm }=m_{A^0}$). As for the lightest Higgs boson mass ($m_{h^{0}}$), we have fixed it to 100 GeV. This value was chosen as it represents the maximum $m_{h^{0}}$ that respects both theoretical and experimental constraints. Additionally, this choice ensures that the *Z* boson remains off-shell across all selected points. Regarding the parameter $|\cos (\beta -\alpha )|$, as we are working within the IH scenario, it must take a value very close to 1. Throughout our study, $|\cos (\beta -\alpha )|$ is varied between 0.8 and 1. Furthermore, to ensure theoretical and experimental feasibility for all points, we have taken $2.5<\tan \beta <10$.

**Fig. 3:**
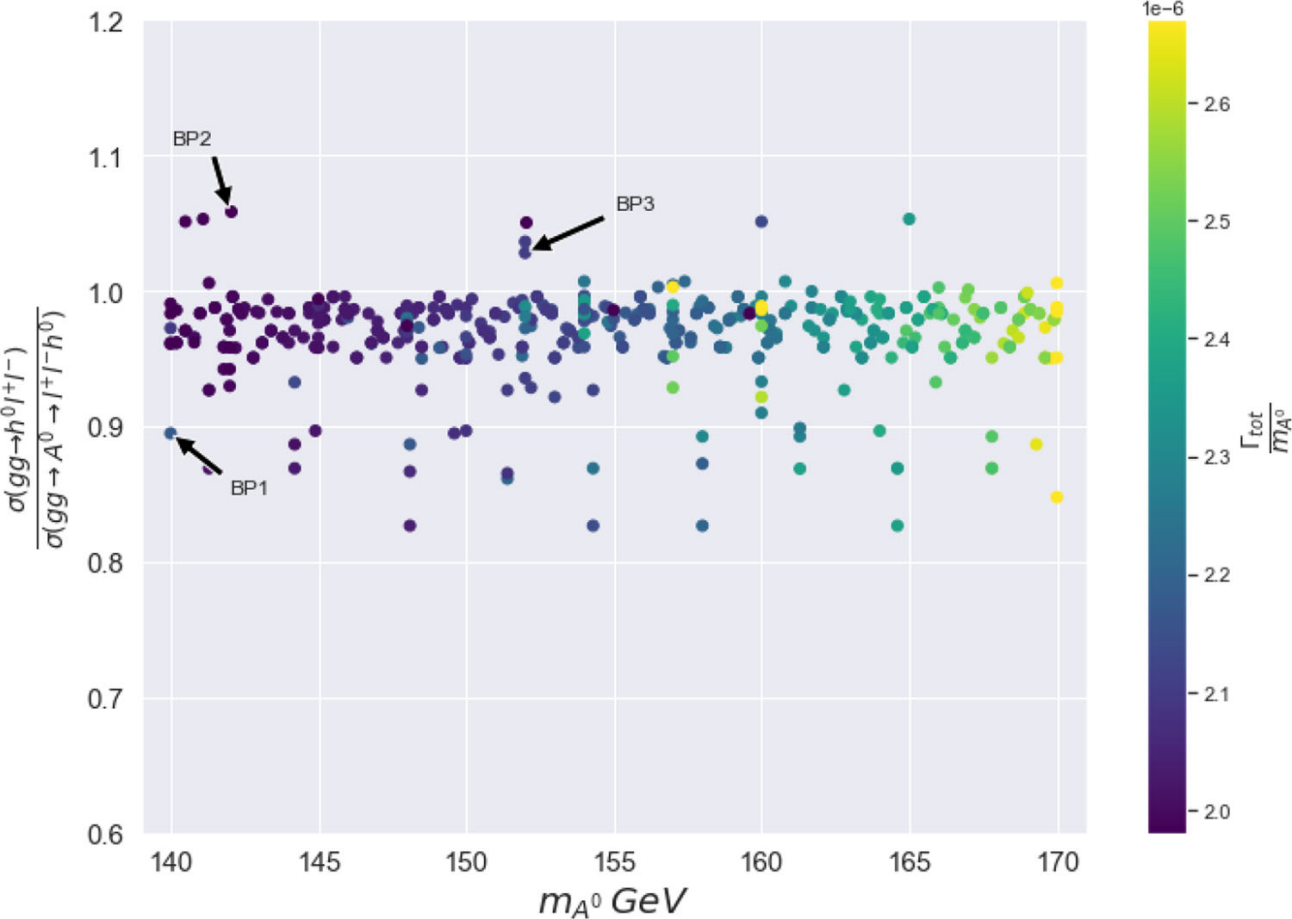
Values of the ratio $\frac{\sigma (gg\rightarrow h^{0}l^{+}l^{-})}{\sigma (gg\rightarrow A^{0}\rightarrow h^{0}l^{+}l^{-})}$ mapped against $m_{A^{0}}$ and colour graded against $\frac{\Gamma _{A^0} }{m_{A^{0}}}$ for a selection of parameter space points surviving all constraints

In Fig. 3 we evaluate (for some 2HDM points that respect all constraints) the ratio $\frac{\sigma (g g \rightarrow h^{0} Z^*\rightarrow h^{0}l^{+}l^{-})}{\sigma (g g \rightarrow A^0 \rightarrow h^{0} Z^*\rightarrow h^{0}l^{+}l^{-})}$ as a function of $m_{A^0}$, where the denominator is the cross section corresponding to the (triangle) amplitude squared involving only the $A^0$ in the *s*-channel (and in NWA) and the numerator is the full process, the latter including the (triangle) amplitude squared with an *s*-channel (off-shell) $Z^*$ and the amplitude squared of the box diagrams, as well as their relative interferences. For the case of the full process, $A^0$ is allowed to be off-shell as well. The size of the interferences depends on the value (depicted in Fig. 3 as a colour gradient) of the ratio $\frac{\Gamma _{A^0}}{m_{A^0}}$. One notices in Fig. 3 that the ratio of the cross sections varies from 1.06 to 0.85 for the chosen points and there is only a mild correlation with $\frac{\Gamma _{A^0}}{m_{A^0}}$, which in turn highlights the counter-balancing role of the box diagrams on their own (in the positive direction) as well as that of their interference with the $A^0$ diagram (in the negative direction) on the final result (the triangle graphs with $Z^*$ in the *s*-channel are generally negligible because of the Landau–Yan theorem [38, 39]). Note that this difference ($+6\% \rightarrow -15\%$) between the cross section for the full process and the factorised one in NWA at the inclusive level in the 2HDM Type-I in IH with a light $m_{h^0}$ is somewhat higher than what was found in a previous work for the same process in the 2HDM Type-II in NH for SM-like Higgs production [8], where differences were confined to the percent level (see also [40]). We further note that, although the same computation (i.e., the cross section of the full process) as the one performed here was also done in [8], the emerging phenomenology is rather different. This is because the size of the $A^0\rightarrow h^0 Z^*$ cross section in the 2HDM IH scenario of the present work is much larger than that in [8] and, hence, the relative magnitude of the interferences is greater.^3^ We also emphasise that in Ref. [8] no comparison between the factorised and full results was made.

**Table 2:** The input values for the parameters defining our three BPs and corresponding cross sections in pb, for all of these we have $\cos (\beta -\alpha ) =1$, $m_{h^0}=100$ GeV and $m_{H^{\pm }}=m_{A^0}$. (Also recall that $m_{H^0}=$ 125 GeV)

BP	$m_{A^{0}}$	$\tan \beta$	$\sigma (gg\rightarrow A^{0}\rightarrow h^{0}l^{+}l^{-})$	$\sigma (g g \rightarrow h^{0} l^{+} l^{-})$
1	140	4.8	0.01296	0.01168
2	142	4.7	0.01458	0.01543
3	152	4	0.03859	0.03964

We now select three Benchmark Points (BPs), which are labelled as BP1, BP2 and BP3 in Fig. 3, for kinematical analysis. The ratio $\frac{\sigma (gg\rightarrow h^{0}l^{+}l^{-})}{\sigma (gg\rightarrow A^{0}\rightarrow h^{0}l^{+}l^{-})}$ is approximately 0.9, 1.06 and 1.03 for BP1, BP2 and BP3, respectively. The 2HDM input parameters and cross sections for the full and factorised process for the three BPs are given in Table 2.

**Fig. 4:**
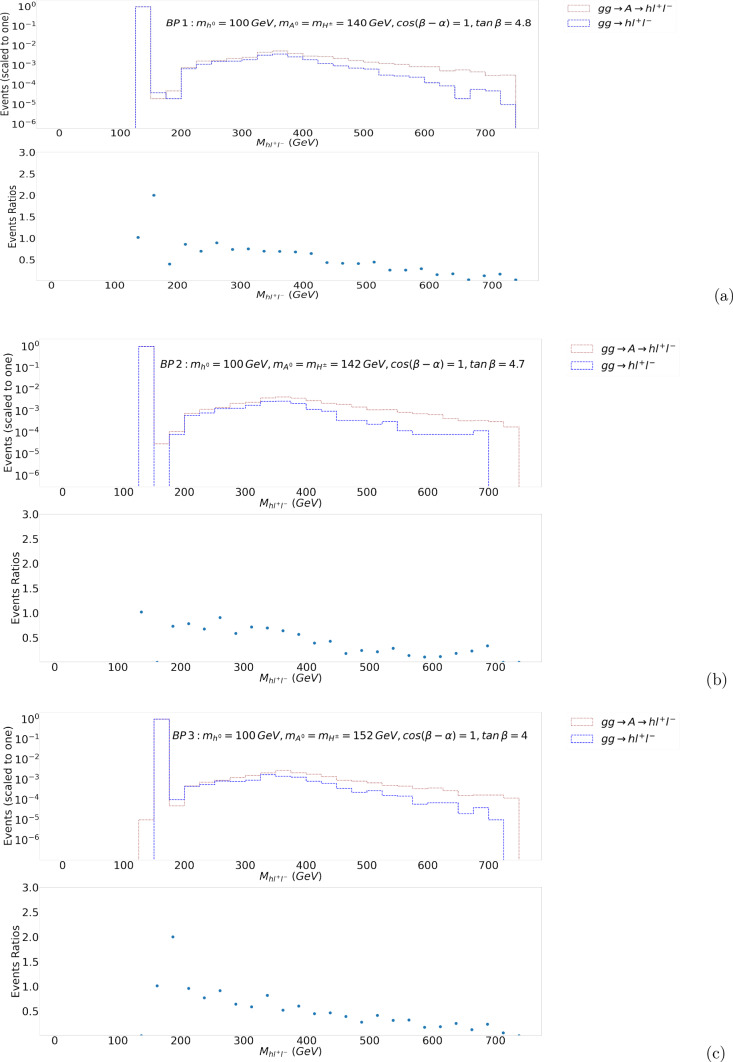
The $M_{h^{0} l^{+}l^{-}}$ histograms (normalised to 1) for BP1 (**a**), BP2 (**b**) and BP3 (**c**) (main frames) together with their corresponding ratios (sub-frames)

The invariant mass distributions for $h^0 l^+l^-$ events of these BPs are given in Fig. 4. The presence of the additional diagrams in the full process $gg\rightarrow h^0l^+l^-$ causes a difference from the results that are obtained for the factorised process $gg\rightarrow A^0\rightarrow h^0l^+l^-$. In Fig. 4, the spectra are normalised to 1 (so that we are tracking the different shapes of the two processes) and we have sampled $M_{h^0l^+l^-}$ over a bin width of 25 GeV. In the case of BP1, the $A^0$ peak height is essentially the same for the two processes, with the factorised approximation being lower than the full result and the former taking over the latter already before the loop threshold at $\approx 2m_t$. For BP2, the $A^0$ peak height is again approximately the same for the two processes while beyond the peak the $A^0$ process dominates (especially so starting from the aforementioned $2m_t$ value) (Notice here a strong negative interference in the full process just beyond $M_{h^0\,l^+l^-}=m_{A^0}$.) In BP3, again the $A^0$ peak height is essentially the same, with the full process being larger just beyond it and subleading far beyond it, with respect to the factorised one. By looking at the ratios of the heights of the histograms, it is clear that differences between the two approaches at the differential level can be much bigger than at the integrated level (recall Table 2), as the difference can be up to several tens of percent in either direction. Furthermore, such effects can have different shapes, so as to alter (unpredictably) the yield expected from a naive approach that probes the $h^0l^+l^-$ final state under the hypothesis that only the process $gg\rightarrow A^{0}\rightarrow h^{0}l^{+}l^{-}$ contributes, as is customarily assumed in typical experimental analyses. However, as can be seen from Fig. 4, the fraction of events that is away from the $A^0$ peak is very small (to be quantified below) and thus we expect that such a difference in the differential distributions (in the event of a discovery of $A^0\rightarrow h^0Z^*$) would only (possibly) start to be manifest with much more integrated luminosity. In fact, the actual size of such effects in an experimental analysis depends on the way the invariant mass of $b{\bar{b}}l^+l^-$ is sampled (here we assume the predominant $h^0\rightarrow b{\bar{b}}$ decay and that the value of $m_{h^0}$ can be reconstructed in actual LHC analyses.)

In existing searches for $A^0$ narrow resonances [47], whereby a tentative hypothesis is made for the value of $m_{A^0}$, so that selection cuts can be optimised accordingly, and the $M_{h^0l^+l^-}$ distribution is sampled in narrow bins, e.g., of 25 GeV (a typical resolution of the $b{\bar{b}}l^+l^-$ system), the effect of the above results for the three BPs would correspond to rescaling the naive expectation stemming from the process $gg\rightarrow A^{0}\rightarrow h^{0}l^{+}l^{-}$ by the amounts $-10\%$, $+6\%$ and $+3\%$ for BP1, BP2 and BP3, respectively (i.e., by the corrections at inclusive level). This is clear from Fig. 4, as most of the $A^0\rightarrow h^0Z^*$ events fall in one bin that contains $M_{A^0}$. Indeed, the percentage of the differential cross section for the full process in Fig. 4 in the 25 GeV bin centred around $m_{A^0}$ is $97.6(98.2)[98.8]\%$, so that only a very small part of it is found in the remaining bins.

However, if a non-resonant search is performed, thereby using for the significance calculation a much wider expanse in such an invariant mass (i.e., with no tentative assumption made on $m_{A^0}$ or $\Gamma _{A^0}$), and the mass intervals used for sampling did not include the $A^0$ peak (e.g., because ‘blind’ selection criteria would eliminate it from the candidate signal sample or such a mass interval is chosen elsewhere from the peak region), overall effects could be drastically different. For example, if one sampled the two processes ${gg\rightarrow h^{0}l^{+}l^{-}}$ and ${gg\rightarrow A^{0}\rightarrow h^{0}l^{+}l^{-}}$ over the mass intervals 200 GeV $< M_{h^0\,l^+l^-}<$ 400(600)[750] GeV, differences between the two would be $-71(-60.4)[-57.7]\%$ for BP1, $-63(-51.4)[-49.7]\%$ for BP2 and $-64(-55.9)[-54.2]\%$ for BP3.^4^ Clearly, such significant effects should not be surprising: they are indeed consistent with the inclusive cross section results since these bins only contain a small fraction of the total number of $h^0 l^+l^-$ events. However, we suggest that they could start to be evident at high integrated luminosity. This may happen under two circumstances. On the hand, they could emerge following a presumed discovery of the resonant $A^0$ state (at a lower integrated luminosity), whereby one could use the high mass regions of the $M_{h^0l^+l^-}$ distribution to extract its properties, notably, around $2m_t$. On the other hand, in the absence of such a discovery, they could simply manifest themselves as a broad excess above the SM preditions, which may be retroactively be used to search for the $A^0$ state, produced resonantly outside of the sampled $M_{h^0l^+l^-}$ interval.

## Conclusions

In summary, we have described the phenomenology of the $gg\rightarrow h^0Z^*(\rightarrow l^+l^-)$ process at the LHC, in the context of the 2HDM Type-I (within NFC) in the IH scenario, i.e., with $m_{h^0}< m_{H^0}=125$ GeV. We have shown that sizable differences can exist between the naive approach wherein the above process is factorised with the $A^0$ state in NWA (i.e., $gg\rightarrow A^0\rightarrow h^0Z^*$) and the full process in which such a Higgs state can be off-shell and also accounting for all other Feynman diagram structures entering it (and corresponding interferences): i.e., $gg\rightarrow Z^*\rightarrow h^0Z^*$ (through one-loop triangle diagrams) and $gg\rightarrow h^0Z^*$ (through one-loop box diagrams). Such differences vary significantly depending on the kind of analysis which is deployed to search for the CP-odd Higgs state, whether resonant or non-resonant, but in both cases amounting to up to several tens of percent at the differential level while at the integrated level rates are never beyond 10% or so. Hence, in the circumstances, a redefinition of the signal is necessary, so as to also capture all such effects entering the cross section involving $A^0$ production and decay, alongside the use of a suitable computational framework.

Indeed, in order to aid the experimental pursuit of the effects described here, we have released in this paper several BPs representative of parameter space configurations enabling sensitivity to what are the most effective (i.e., resonant, where the majority of the signal would manifest itself) searches for $h^0Z^*(\rightarrow l^+l^-)$ production and decay as well as potentially visible tail effects (where only a minority of the signal would be present) from such a process appearing in non-resonant searches (i.e., those not specifically optimised to the model and/or process considered here). Finally, we will eventually make the computing tools developed here also available in the public domain.

## Data Availability

Data will be made available on reasonable request. [Author’s comment: The datasets generated during and/or analysed during the current study are available from the corresponding author on reasonable request].

## Footnotes

1 We have tested our population of surviving scan points (see below) against the most recent HiggsTools implementation of such constraints [14] and noticed very minimal differences, altogether not affecting the parametric scenarios we tested.

2 Next-to-LO (NLO) corrections in QCD exist [30–36], which – while being sizable inclusively – do not alter significantly the $\sqrt{\hat{s}}$ distribution (which will be of concern here). No NLO EW corrections have been computed to date.

3 The effects of interference between triangle and box topologies is also well known for single SM-like Higgs production in all di-boson final states [41–46].

4 Note that there are negative interferences in the mass region between $m_{A^0}$ and $m_{h^0}+m_Z$ which have deliberately not been included in our $M_{h^0l^+l^-}$ sampling and could make such differences even more dramatic.

## Code Availability

Code/software will be made available on reasonable request. [Author’s comment: The code/software generated during and/or analysed during the current study is available from the corresponding author on reasonable request].
